# Supplementary material for: A web server for comparative analysis of single-cell RNA-seq data
Source: Nat Commun. 2018 Nov 13;9:4768. doi: 10.1038/s41467-018-07165-2 (PMC6233170; doi:10.1038/s41467-018-07165-2)
Supplement: Supplementary file 1 — Supplementary Information [file 41467_2018_7165_MOESM1_ESM.pdf]

Supplementary Information for
A web server for comparative analysis of single-cell RNA-seq data

Amir Alavi[1,†], **Matthew Ruffalo**[1,†], **Aiyappa Parvangada**[1], **Zhilin Huang**[1], and **Ziv Bar-Joseph**[1,2,*]

[1]Computational Biology Department
[2]Machine Learning Department
School of Computer Science, Carnegie Mellon University, Pittsburgh, PA 15213

[†]Equal contribution
[*]Corresponding author: zivbj@cs.cmu.edu

Supporting Website: https://scquery.cs.cmu.edu

Supplementary Figures

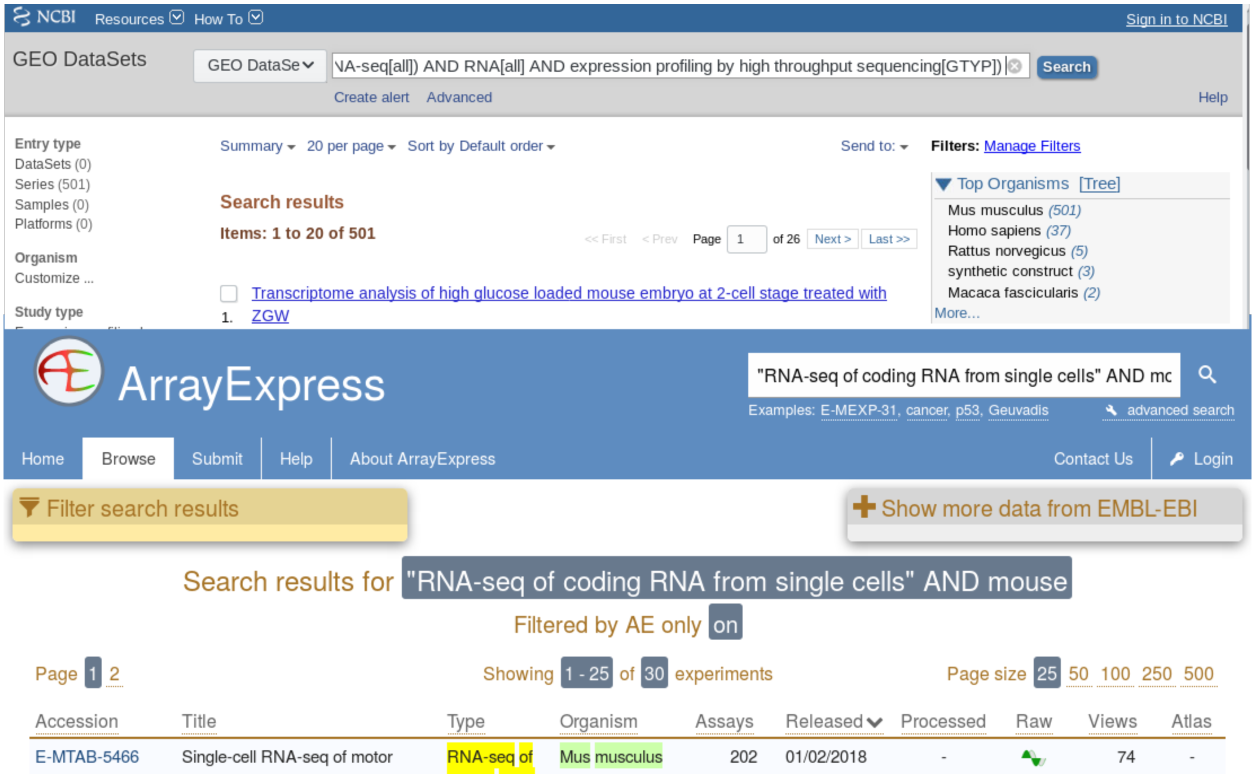

Supplementary Figure 1: **Example data query.** Our queries to the NCBI GEO and ArrayExpress systems, selecting mouse single-cell RNA-seq data.

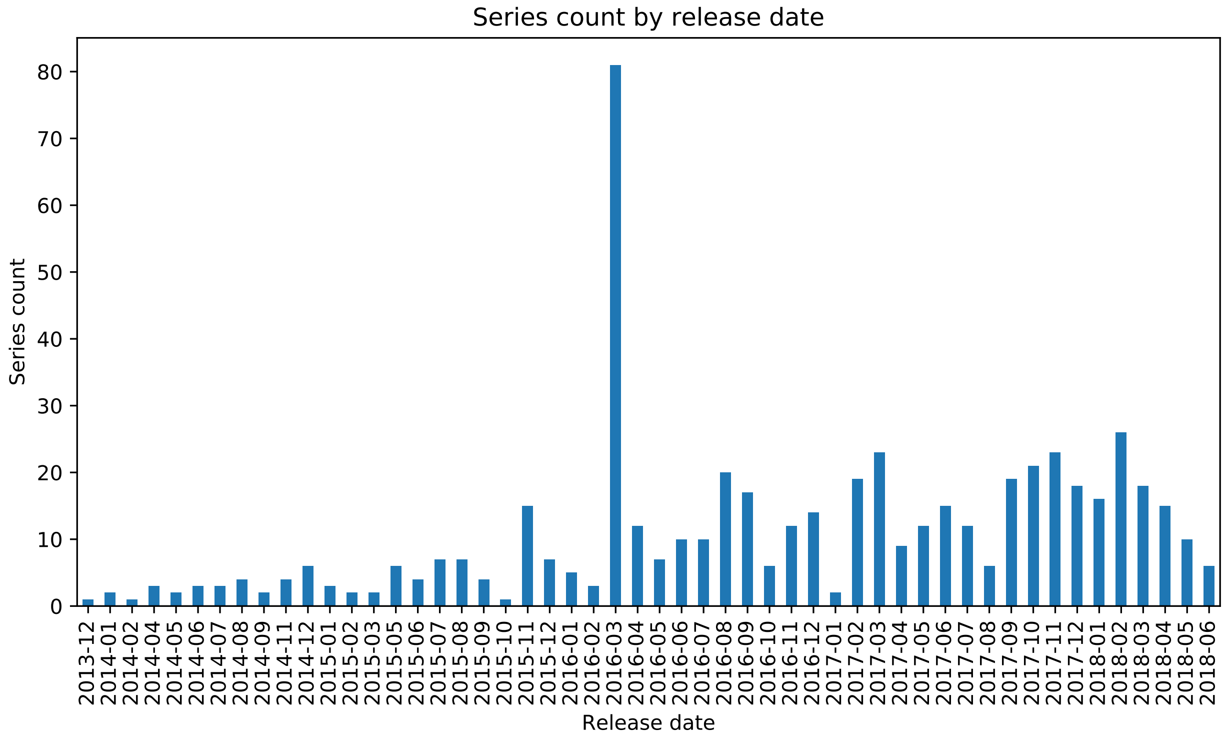

Supplementary Figure 2: **Monthly study count available on GEO and ArrayExpress.** Number of studies released each month in GEO and ArrayExpress.

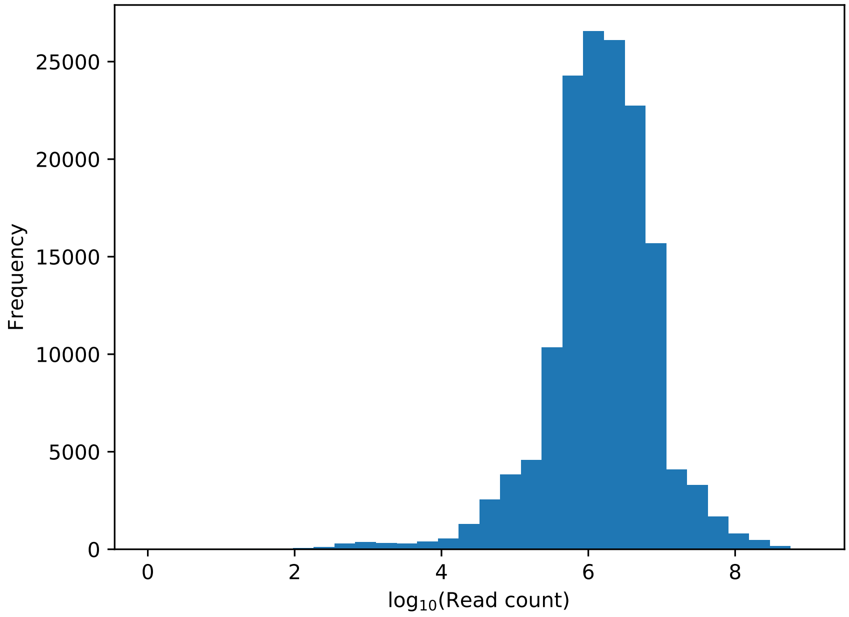

Supplementary Figure 3: **Distribution of reads in available raw data.** Number of RNA-seq reads in each available series/cell, across all studies.

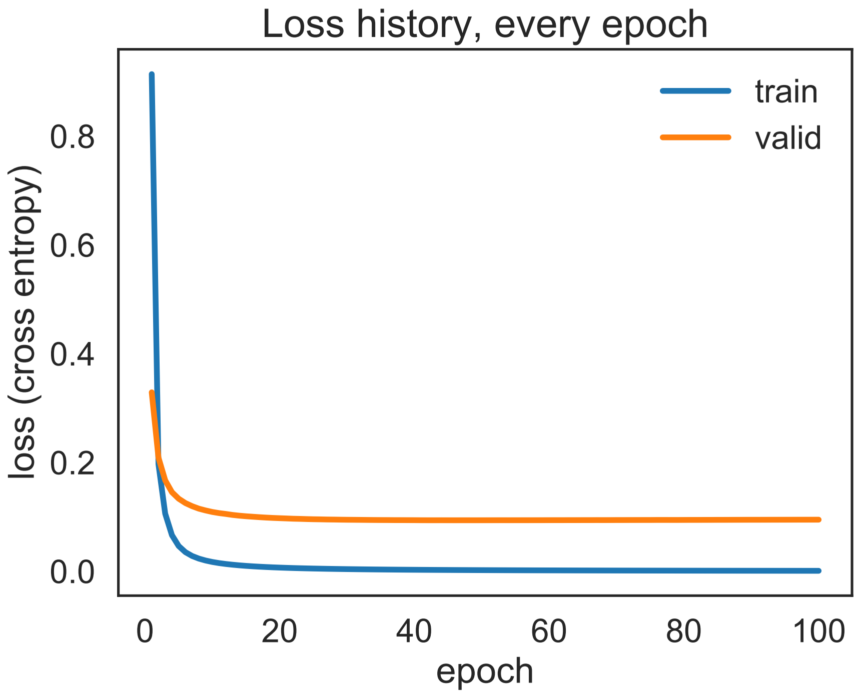

Supplementary Figure 4: **Neural network training history.** Training and validation loss exhibiting convergence within 100 epochs for the "PT dense 1136 100" model in Figure 3. The weights saved are those of the first iteration after which validation loss no longer improves.

Model	# Parameters	Train set size	Mini-batch size	Training time
PT dense 1136 100	23,406,245	36,473	256	3 mins 54 secs
PT dense 1136 500 100	23,911,145	36,473	256	3 mins 51 secs
triplet PT ppitf 1136 500 100	3,116,269	72,000	72	31 mins 5 secs
triplet hierarchical GO	1,123,151	72,000	72	1 hr 57 mins
Siamese PT dense 1136 500 100	23,906,600	110,117	256	27 mins 13 secs
Siamese PT ppitf 1136 100	2,611,369	110,117	256	41 mins 18 secs

Supplementary Table 1: **Neural network training times.** Number of parameters and time to train for each of the neural network model types from Figure 3. "PT" indicates that the model had been pretrained using the unsupervised strategy (Supporting Methods). Numbers after the model name indicate the hidden layer sizes. "hierarchical GO" refers to the GO-based architectures (Supporting Methods). All models were trained for 100 epochs on a machine with two Intel(R) Xeon(R) E5-2620 v3 @ 2.40GHz CPUs and an Nvidia GeForce GTX 1080 GPU.

t-Test: Paired Two Sample for Means

	PT dense 1136 100	*PCA 100*
Mean	0.576379804	0.5440947
Variance	0.207874104	0.18939181
Observations	2330	2330
Pearson Correlation	0.845531936	
Hypothesized Mean Difference	0	
df	2329	
t Stat	6.272445056	
P(T<=t) one-tail	2.11189E-10	
t Critical one-tail	1.645508148	
P(T<=t) two-tail	4.22378E-10	
t Critical two-tail	1.960983084	

Supplementary Table 2: **Paired t-test comparing PT dense 1136 to PCA 100.** The tested dataset is the full set of 2330 cells in our query set. Each observation is the Average Flexible Precision score on a query cell. We see that our best neural embedding model outperformed PCA 100 ($p = 4.224 \times 10^{-10}$.)

	# in query	9	42	37	45	250	16	
	# in database	207	273	50	250	340	161	
**Architecture type**	**Model**	**fibroblast**	**macrophage**	**blastoderm**	**embryo**	**retina**	**liver**	**weighted average (lowly rep. cell types)**
Cell type classifier	PT dense 1136 100	**1.000**	**1.000**	**0.990**	0.145	0.909	0.129	0.468
	PT dense 1136 500 100	**1.000**	**1.000**	0.973	0.091	0.909	0.248	0.460
Triplet	PT (frac active) ppitf 1136 500 100	**1.000**	0.980	0.541	0.493	0.941	0.385	0.508
	PT (val loss) ppitf 1136 500 100	**1.000**	0.998	0.478	**0.640**	**0.942**	**0.481**	0.512
Siamese	PT dense 1136 500 100	0.991	0.964	0.660	0.258	0.896	0.110	**0.791**
	PT ppitf 1136 100	**1.000**	0.874	0.730	0.082	0.870	0.158	0.419
N/A	PCA 100	0.832	0.993	0.660	0.167	0.901	0.097	0.454
	Original data	0.279	0.826	0.262	0.146	0.797	0.200	0.368

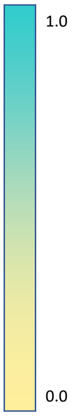

Supplementary Figure 5: **Retrieval testing results for lowly represented cell types in our database.** Results presented in a similar manner as Figure 3, except that here we only show the results of those cell types with less than 1% of total training set population (36,473 cells). The final column is the weighted average score over all such "lowly represented" cell types (some not shown in table due to space), and the weights are the number of such cells in the query set.

Architecture type	Model	hematop-oietic stem cell	osteocyte	neuron	neural	embryo	retina	skin epidermis	weighted average (higly rep. cell types)
	# in query	160	45	162	81	45	250	678	
	# in database	645	481	421	523	250	340	1097	
Cell type classifier	PT dense 1136 100	0.241	0.984	0.942	0.811	0.246	0.903	0.629	**0.649**
	PT dense 1136 500 100	0.196	0.990	0.935	0.526	0.242	0.914	0.480	0.534
Siamese	PT dense 1136 500 100	0.125	0.883	0.846	0.666	0.233	0.895	0.499	0.543
	PT ppitf 1136 100	0.053	0.695	0.242	0.672	0.267	0.828	0.307	0.335
N/A	PCA 100	0.369	0.959	0.701	0.747	0.184	0.904	0.485	0.504
	Original data	0.130	0.974	0.539	0.824	0.146	0.797	0.062	0.122

Color scale: 1.0 – 0.0

Supplementary Figure 6: **Retrieval testing results for reprocessed data only.** Retrieval testing results of various architectures, as well as PCA and the original (unreduced) expression data, trained on only the data that we processed ourselves. "PT" indicates that the model had been pretrained using the unsupervised strategy (Supporting Methods). Numbers after the model name indicate the hidden layer sizes. For example, "dense 1136 500 100" is an architecture with three hidden layers. The final column is the weighted average score over those cell types with at least 1000 cells in the database (some not shown in table), and the weights are the number of such cells in the query set.

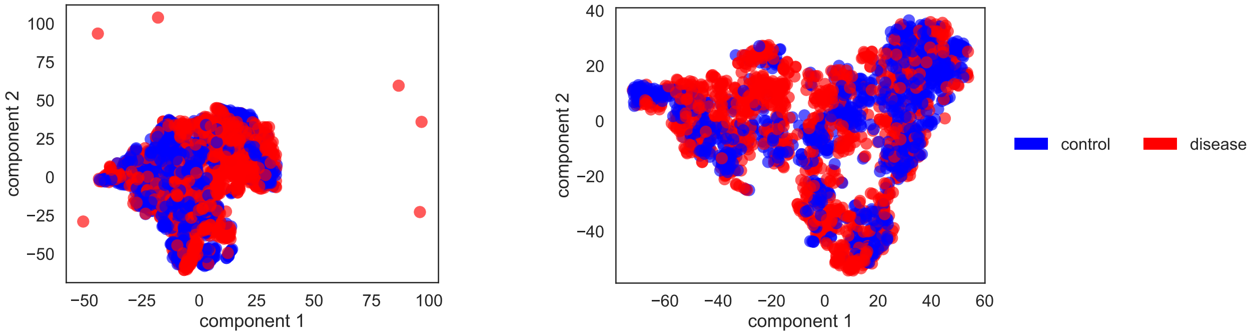

(a) original data

(b) PT dense 1136 100

Supplementary Figure 7: **t-SNE visualizations of mouse brain query cells.** (a) Two component t-distributed stochastic neighbor embedding (t-SNE) of the query cells from their original 20,499 dimensions (genes), colored by disease status. (b) Two component t-SNE of the query cells from the 100 dimensional neural embedding via the "PT dense 1136 100" model.

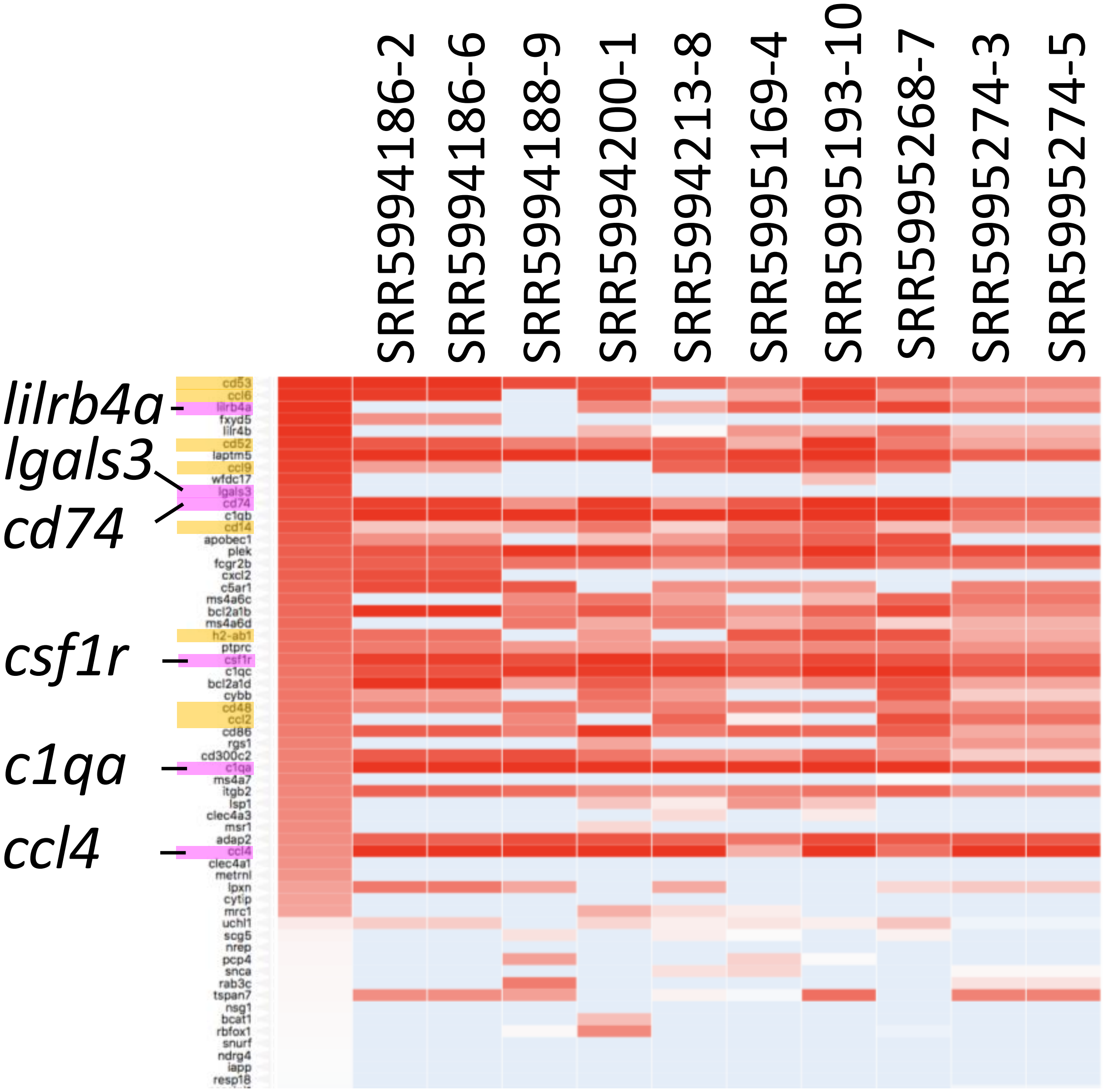

Supplementary Figure 8: **Gene expression within mouse neurodegeneration dataset, late response cells.** This figure is a crop from a screenshot of an scQuery session. logRPKM expression of the macrophage-specific genes (Methods) within a cluster of late-stage (6 weeks after p25 induction) neurodegenerative cells enriched for "macrophage" labeling by our retrieval server. The leftmost column is the average logRPKM among our database macrophage cells. Genes highlighted in magenta were also found to be up-regulated in the original study [1]. Genes highlighted in yellow are additional genes related to immune response that are up-regulated in the query cells. Genes highlighted in yellow in order of top to bottom: *Cd53, Ccl6, Cd52, Ccl9, Cd14, H2-ab1, Cd48, Ccl2*

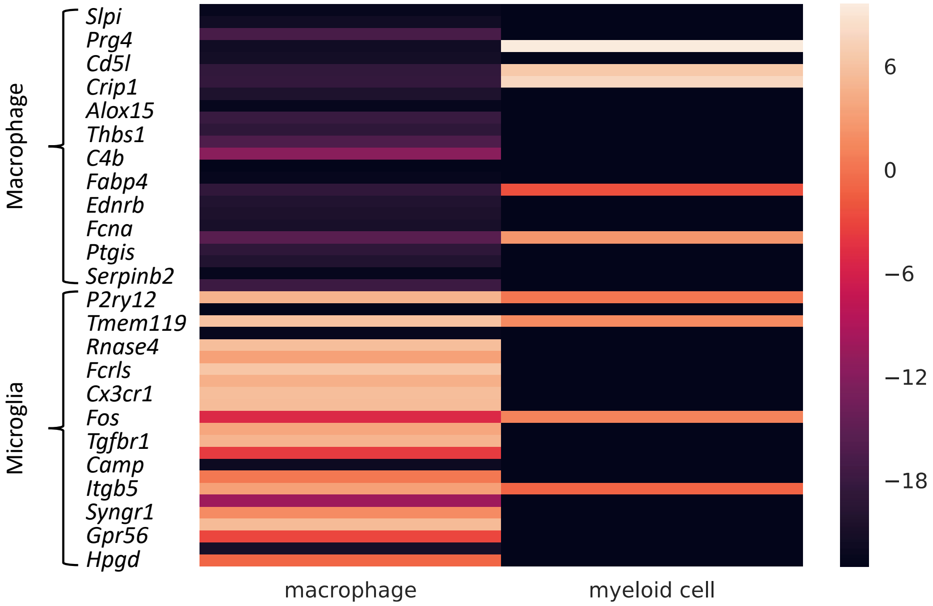

Supplementary Figure 9: **Marker gene expression in mouse brain immune cells.** Expression ($\log_2$(RPKM)) of marker genes [2] for macrophages and microglial cells for all cells we classified as "macrophage" or "myeloid" cell.

Algorithm 1: Cell type-specific gene list construction algorithm. The outermost loop of the algorithm is over different cell types. If there exists multiple studies of significant size ($> m$) that have profiled a particular cell type, then there is an inner loop that does a differential expression analysis for each such study. Otherwise, the algorithm will try to do a single differential expression analysis by pooling the cells of this type from the various studies. If there were multiple studies, then the final list of DE genes for a cell type is created from a meta-analysis of the DE genes from each study (Supporting Methods).

```
input  : D = matrix of gene expression values (preprocessed as described in Supporting Methods)
         m = minimum cells in a DE experiment
         DEModule(goup1, group2) = Differential Expression analysis module
output: CellTypeGenes = Lists of cell type-specific differentially expressed genes

CellTypeGenes ← {∅}
foreach cell type C ∈ D do
    DEResults ← {∅}
    D′ ← only cells of type C from D
    S ← set of studies in D′
    if ∃ a study s ∈ S that contains at least m cells of type C then
        foreach study s that satisfies the above condition do
            a ← SampleXFromY(m, D′_s)
            b ← SampleXFromY(m, D)
            DEResults ← DEResults ∪ DEModule(a, b)
        end
    else if |D′| >= m then
        (If there doesn't exist at least one study for C with at least m cells, then
        we try to combine all of C's studies into a single group)
        a ← SampleXFromY(m, D′)
        b ← SampleXFromY(m, D)
        DEResults ← DEResults ∪ DEModule(a, b)
    else
        continue
    end

    CellTypeGenes ← CellTypeGenes ∪ MetaAnalysis(DEResults)
end
```

Number of Queries	Linear Scan (secs)	NMSLib (secs)	Accuracy
100	0.457	0.001967	0.9819
500	2.417	0.005926	0.9543
1000	4.447	0.013977	0.9459

(a) NMSLB

Number of Queries	Linear Scan (secs)	ANNoY (secs)	Accuracy
100	0.457	0.028205	0.9673
500	2.417	0.137404	0.97726
1000	4.447	0.278778	0.94861

(b) ANNoY

Number of Queries	Optimal Number of probes	Linear Scan (secs)	FALCONN (secs)	Accuracy
100	16	0.457	0.028205	0.9673
500	31	2.417	0.137404	0.97726
1000	346	4.447	0.278778	0.94861

(c) FALCONN

Supplementary Table 3: **Approximate nearest neighbor search method comparison.** Nearest neighbor search benchmarking results for **(a)** NMSLB, **(b)** ANNoY, and **(c)** FALCONN.

Supplementary Methods

Labeling of single cell experiments using Cell Ontology terms

We use all available labels in the Cell Ontology to assign each single cell experiment to a set of ontology terms. We consider all cell labels and synonyms, and adjust each label/synonym before performing text matching against single cell experiment metadata. We strip the " `cell`" or " `cells`" suffixes, if present and if the cell descriptor consists of two or more characters without those suffixes. For example, "`b cell`" is used as-is, but "`brain cell`" is converted to "`brain`" before performing text searching. We build a regular expression for each adjusted label/synonym, replacing "`{term}`" with the appropriate label/synonym:

```
(-|\b)({term})(s?)(-|\b)
```

This allows every term, with or without a "`s`" suffix, to be matched with adjoining word boundaries or "`-`" characters.

We use the 'source,' 'cell type,' and 'cell ID' fields in GEO and ArrayExpress to assign a set of Cell Ontology terms to each single cell experiment. We collect all unique values in these fields across all single cell experiments, and test whether each value matches the regular expression for each ontology term name/synonym. We then reduce the set of terms for each experiment, removing any term that is an ancestor of another. For example, the cell label "Bone marrow cells" is assigned the following terms:

- CL:0000000 cell
- CL:0000137 osteocyte
- CL:0001035 bone cell
- CL:0002092 bone marrow cell
- UBERON:0001474 bone element
- UBERON:0002371 bone marrow
- UBERON:0002481 bone tissue

Removing redundant terms leaves only "CL:0000137 osteocyte" and "CL:0002092 bone marrow cell".

Triplet architectures trained with batch-hard loss

Following the same motivations as siamese networks, triplet networks also seek to learn an optimal embedding but do so by looking at three samples at a time instead of just two as in a siamese network. The triplet loss used by Schroff et al. considers an point (anchor), a second point of the same class as the anchor (positive), and a third point of a different class (negative) [3].

$$\text{Triplet Loss} = \sum_{i=1}^{T} \left[(D^i_{a,p})^2 - (D^i_{a,n})^2 + \alpha \right]_+ \tag{1}$$

Where:

$\boldsymbol{T}$ is the set of all training examples (triplets of data points)

$\boldsymbol{D_{a,p}}, \boldsymbol{D_{a,n}}$ are the Euclidean distances computed by the network between the embeddings of the anchor point and the positive point, and the embeddings of the anchor point and the negative point, respectively

$\boldsymbol{\alpha}$ is a margin hyperparameter

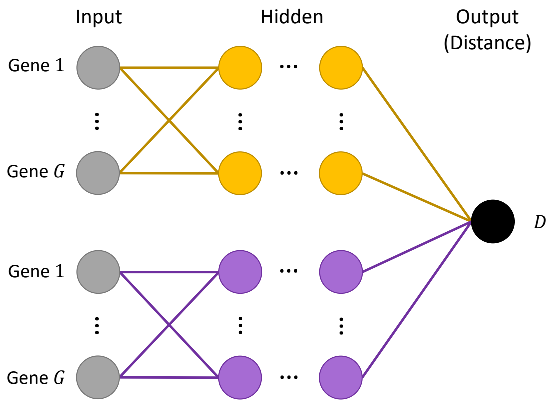

Supplementary Figure 10: **Siamese neural network configuration.** Weights are shared between the yellow and the purple subnetworks. Data is fed in pairs with one item in the pair going through the yellow subnetwork, and the other through the purple subnetwork. Triplet networks operate in a similar manner, except that three points are fed through three subnetworks (again, shared weights).

When training a siamese or triplet network, it is very important to select pairs/triplets that are difficult enough so that the network learns and converges [3] . A straightforward approach to mine hard examples would involve periodically pausing training, embedding all data points using the current weights, calculating pairwise distances among all of these points in the embedded space, and selecting new examples based on this. This is computationally expensive and is prone to selecting pairs that are too difficult (outliers), so here we have opted to mine challenging pairs in an online fashion by selecting from points within a mini-batch [3, 4]. Specifically, we use Hermans et al.'s "batch hard" loss, shown in Equation 2 [4]. The loss is calculated over a mini-batch, which is constructed by first sampling P cell types (labels) and then sampling K cells of each type.

$$\text{Batch Hard Loss} = \sum_{i}^{P} \sum_{a}^{K} \left[m + \max_{p=1,\ldots,K} D\left(f(x_a^i), f(x_p^i)\right) - \min_{\substack{j=1,\ldots,P;j\neq i \\ n=1,\ldots,K}} D\left(f(x_a^i), f(x_n^j)\right) \right]_+ \tag{2}$$

Where:

$\boldsymbol{f}$ is the neural network (an embedding function)

$\boldsymbol{x_c^t}$ is the cth cell of type t in the mini-batch

$\boldsymbol{D(a,b)}$ is the Euclidean distance between vectors $\boldsymbol{a}$ and $\boldsymbol{b}$

$\boldsymbol{m}$ is a margin hyperparameter

For each anchor point a in the mini-batch, the max term in Equation 2 finds the hardest positive pair, while the min term finds the hardest negative pair.

Unsupervised neural network pretraining

While all of our neural embedding models are trained in a supervised fashion (for cell-type classification or embedding optimization), we also used unsupervised training to initialize the weights for these models, and this was shown to improve performance (Results, Figure 3). We have shown in prior work that using a denoising autoencoder (DAE) for unsupervised pretraining can improve performance [5]. Here we extend this to using stacks of DAEs, which are each individually trained in a process called "greedy layer-wise pretraining" to pretrain each layer of our neural network architectures [6, 7].

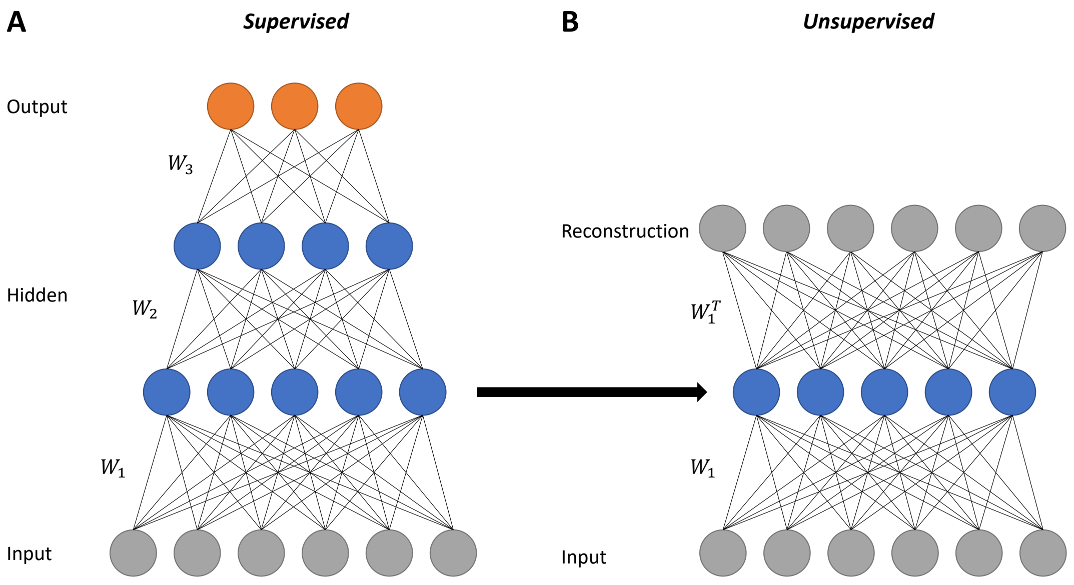

Supplementary Figure 11: **Illustration of one stage of greedy layer-wise unsupervised pretraining of a neural network.** W_1, W_2, and W_3 are weight matrices. **(A)** A supervised neural network with two hidden layers to be pretrained. **(B)** An unsupervised DAE that has the the supervised network's first hidden layer as its middle layer. This DAE is trained to minimize the mean squared error between its reconstruction and the original input data (prior to corruption with noise). After training this DAE, its weights can be used as initial values for the weights in the supervised network.

Here we illustrate the process with an example of how we would pretrain a neural network with an input layer, two hidden layers, and an output layer (Supplementary Figure 11a). Supplementary Figure 11b shows how the first hidden layer is pretrained. The first hidden layer becomes the hidden layer in a DAE, with a "reconstruction" layer of the same dimensionality as the input added on top. Gaussian noise is added to each sample and fed through this DAE, and the training objective is to minimize the mean squared error between the reconstructed output and the clean, uncorrupted samples. After training this DAE, the weights can be used to initialize the first hidden layer in our supervised neural network. This process is repeated for the other hidden layers, where all weights below the layer of interest are fixed, and the input is first fed forward through these fixed weights to get a new representation, and then the corrupting noise is added to this new representation for training the DAE of the current layer.

Cell-type similarity calculation

Rather than rely on distances based on the ontology graph structure, we computed cell type similarities with a data driven approach by using term co-occurrences in PubMed. For each pair of cell types in our ontology, we queried PubMed for articles which had both of the terms in the title or abstract texts.

We then define S_i as the number of articles in which term i occurred in, and $P_{i,j}$ as the number of articles in which terms i and j co-occurred in. We additionally define a binary matrix $\boldsymbol{M}$ of shape (number of ontology terms, number of documents), where $M_{i,j} = 1$ if term j occurred in document i.

The similarity between two terms can then be computed as a cosine similarity:

$$\begin{aligned} D_{i,j} &= cos(\boldsymbol{M_i}, \boldsymbol{M_j}) \\ &= \frac{\boldsymbol{M_i} \cdot \boldsymbol{M_j}}{|\boldsymbol{M_i}||\boldsymbol{M_j}|} \\ &= \frac{P_{i,j}}{\sqrt{S_i S_j}} \end{aligned}$$

The above measure is not symmetric, and a symmetric version can be defined as:

$$D_{i,j} = \frac{P_{i,j}/S_i + P_{i,j}/S_j}{2}$$

For our neural embedding models that rely on computing distances between cell types (siamese architectures), we use the symmetric version of the cell type similarities. We use the non-symmetric version to evaluate all of our neural embedding models (*i.e.* to compute the MAFP values in our retrieval analysis).

Mean average flexible precision

We employ a modified version of mean average precision (MAP) called mean average flexible precision (MAFP) in order to evaluate the retrieval performance of our dimensionality reduction models. Traditional average precision operates on ranked lists (*e.g.* a list of nearest neighbor cells in a retrieval database) by calculating the precision at exact-match cutoffs in the list, and then taking the mean of these. This requirement is too restrictive for proper evaluation here, as a binary hit or miss criterion would not allow a list containing similar cell types to be valued higher than lists with less relevant cell types. Instead, average flexible precision is computed by summing the cell-type similarities between the query cell and each retrieved result and dividing by the length of the list at a particular depth, and finally taking the average of these values across all depths in the list.

Missing data imputation

Our uniform preprocessing pipeline requires the raw reads data for each study. For certain studies, this is not available, and only the RPKM values processed by the authors of the original study are present. We would still like to integrate this data into our database, which requires us to impute the values of the expression of missing genes.

We conduct missing value imputation with a k-nearest neighbors-based approach, similar to procedures in prior work [5, 8]. We ignore cells which have more than 20% of our gene set missing. After filtering those cells out, we impute the remaining values for each study independently. For each study, in order to compute nearest neighbor genes within the study we first fill the missing values with the median expression value in each cell. We then calculate the euclidean distance matrix of the genes in the study (where each gene is represented by a vector of its expression values in each cell). Finally, the value of each missing gene is imputed with the average expression of its 10 nearest neighbor genes within each cell.

Preprocessing strategies for differential expression analysis

Prior to conducting differential expression analysis, we filter out low-quality genes and cells. To filter out lowly-expressed genes, we first calculate the average reads of each gene within each cell type. We then calculate an overall average for each gene by taking the average of its cell-type specific average expressions. We then only keep genes that have an overall average value that is above a cutoff of 50. This resulted in the removal of 4180 out of 20499 genes.

To filter out cells, we remove cells with fewer than 1.8×10^3 detected genes, which is the default procedure in the tool we have chosen to use for DE analysis, SCDE [9]. This resulted in the removal 732 cells out of 23982 cells. We note that the 23982 cells are a subset of our total database, as we only conduct DE analysis for cell types with a minimum of 75 cells.

Meta-analysis of differential expression experiments

For cell types that have data from multiple studies, we conduct separate DE analysis for each of those studies, and then combine the results to obtain a single list of differentially expressed genes with their p-values. To do this, we take the maximum FDR adjusted p-value for each gene across the studies as its final p-value. Our list of significant DE genes for a particular cell type is then taken as the set of genes with final p-values below a threshold of 0.05.

Additionally, we only keep the DE genes that had a consistent sign (positive or negative) $\log_2$ fold-change across the multiple DE analysis that were done for the particular cell type.

Gene Ontology enrichment analysis

We took the output of Supplementary Algorithm 1 (a list of differentially expressed genes for each cell type) and filtered to keep the top 50 most significant (by FDR adjusted p-value) up-regulated genes. We then used this ordered set of genes as our query for GO enrichment analysis using g:Profiler [10]. We specified that g:Profiler should take the order of the query list into account (more significantly up-regulated genes are more informative) [11]. Other parameters of our analysis were organism="mmusculus", that Benjamini-Hochberg multiple testing correction be used, and that "GO: Biological Process" should be used as the term source. We also supplied a list of 16968 genes to be used as statistical background. These background genes are the subset of our 20499 genes that remain after we filtered out lowly expressed genes in the preprocessing step.

**Cell Type**	retina	
**Term ID**	UBERON:0000966	
# of experiments	2	
**GO ID**	**Name**	**p-value**
GO:0050877	nervous system process	7.63e-09
GO:0050953	sensory perception of light stimulus	7.63e-09
GO:0007601	visual perception	7.63e-09
GO:0007423	sensory organ development	8.69e-09
GO:0060041	retina development in camera-type eye	8.69e-09
GO:0007600	sensory perception	4.09e-08
GO:0001654	eye development	3.96e-07
GO:0003008	system process	6.67e-07
GO:0009584	detection of visible light	6.86e-07
GO:0043010	camera-type eye development	1.23e-06

(a) SCDE

**Cell Type**	retina	
**Term ID**	UBERON:0000966	
# of experiments	2	
**GO ID**	**Name**	**p-value**
GO:0045721	negative regulation of gluconeogenesis	3.03e-02
GO:0032379	positive regulation of intracellular lipid tr...	3.03e-02
GO:0032385	positive regulation of intracellular choleste...	3.03e-02
GO:0061179	negative regulation of insulin secretion invo...	3.03e-02
GO:1905600	regulation of receptor-mediated endocytosis i...	3.03e-02
GO:0010888	negative regulation of lipid storage	3.03e-02
GO:0032382	positive regulation of intracellular sterol t...	3.03e-02
GO:1905602	positive regulation of receptor-mediated endo...	3.03e-02
GO:0045475	locomotor rhythm	3.03e-02
GO:0090118	receptor-mediated endocytosis involved in cho...	3.34e-02

(b) limma-voom

Supplementary Table 4: **Comparison of SCDE (a) and limma-voom (b) when used as "`DEModule`" in Supplementary Algorithm 1**. We conducted the same process as for Table 1 to produce the above two panels: We used GO: Biological Process as the source term set, and used the top 50 up-regulated differentially expressed genes from our cell type-specific gene lists for retina. The top 10 GO terms are shown, sorted by FDR adjusted p-values.

PPI/TF architectures

Since our set of input genes is different, the architectures discussed in Lin et al. could not be used directly here. We used the same transcription factor data as Lin et al. [12]. For our protein-protein interaction data, we use a protein-protein interaction network which integrates known and predicted interactions from multiple databases [13].

Gene Ontology based architectures

We also tested another method to group genes based on the Gene Ontology (GO) [14]. GO is a directed acyclic graph (DAG) whose nodes are gene products (RNA or protein) and whose edges are relationships between these gene products. Along with this graph, there exist curated annotations which associate genes

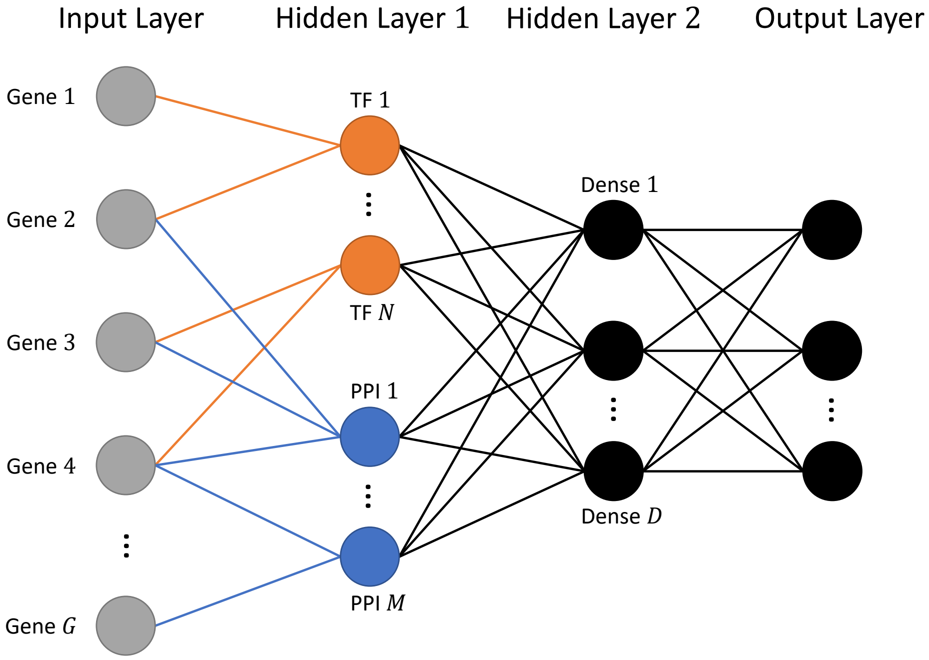

Supplementary Figure 12: **PPI/TF neural network architecture.** Our Protein-protein/protein-DNA (PPI/TF) based neural network architecture, showing sparsely connected layers based on prior biological knowledge.

from specific organisms with each node in the graph. Here we propose that GO's hierarchical structure will enable our models to recognize complicated relationships that most likely exist between genes.

To construct a hierarchical neural network architecture that mirrors the structure of the GO DAG, we first use the published GO annotations for *M. musculus* [15, 16] to associate each of our input genes with a node in GO. Specifically, we choose a particular depth d (distance from a root node) and first associate the genes with only the nodes at this depth in the GO graph. Multiple genes can be associated with the same node. Then we use this grouping of the input genes as the first hidden layer of a neural network. Each node in this hidden layer represents a GO node at depth d with its associated input genes connected to it. The nodes in the next hidden layer will be constructed from GO nodes that have depth d-1. A node in a layer is connected to a node in the layer above if a path exists between their corresponding nodes in the GO graph. We continue this process until the last hidden layer has the desired number of nodes (the size of our reduced dimension). The final result is the network depicted in Supplementary Figure 13.

A key point is that the connections in the network are sparse: a node is only connected to a subset of the nodes in the layer below. This is analogous to the idea of convolutional neural networks (CNNs), in which neurons in a given layer are only connected to a subset of neurons in the layer below (the neuron's "receptive field") [17].

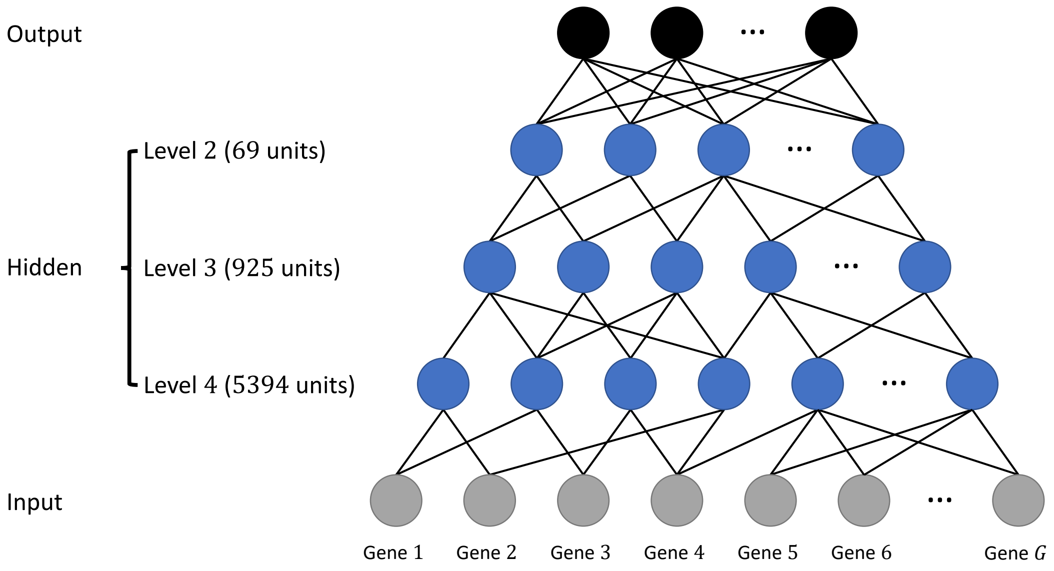

Supplementary Figure 13: **Our Gene Ontology-based neural network architecture.** The connections between the input layer and the first hidden layer, as well a the connections between hidden layers, are sparse and follow connections in the GO DAG. The final hidden layer is fully connected (dense) to the output layer.

Supplementary References

1. Mathys, H. *et al.* Temporal Tracking of Microglia Activation in Neurodegeneration at Single-Cell Resolution. *Cell reports* **21,** 366–380 (2017).
2. Hickman, S. E. *et al.* The microglial sensome revealed by direct RNA sequencing. *Nature neuroscience* **16,** 1896 (2013).
3. Schroff, F., Kalenichenko, D. & Philbin, J. *Facenet: A unified embedding for face recognition and clustering* in *Proceedings of the IEEE conference on computer vision and pattern recognition* (2015), 815–823.
4. Hermans, A., Beyer, L. & Leibe, B. In defense of the triplet loss for person re-identification. *arXiv preprint arXiv:1703.07737* (2017).
5. Lin, C., Jain, S., Kim, H. & Bar-Joseph, Z. Using neural networks for reducing the dimensions of single-cell RNA-Seq data. *Nucleic Acids Research* **45,** e156 (2017).
6. Vincent, P., Larochelle, H., Lajoie, I., Bengio, Y. & Manzagol, P.-A. Stacked denoising autoencoders: Learning useful representations in a deep network with a local denoising criterion. *Journal of Machine Learning Research* **11,** 3371–3408 (2010).
7. Bengio, Y., Lamblin, P., Popovici, D. & Larochelle, H. *Greedy layer-wise training of deep networks* in *Advances in neural information processing systems* (2007), 153–160.
8. Troyanskaya, O. *et al.* Missing value estimation methods for DNA microarrays. *Bioinformatics* **17,** 520–525 (2001).
9. Kharchenko, P. V., Silberstein, L. & Scadden, D. T. Bayesian approach to single-cell differential expression analysis. *Nature methods* **11,** 740 (2014).
10. Reimand, J. *et al.* g: Profiler—a web server for functional interpretation of gene lists (2016 update). *Nucleic acids research* **44,** W83–W89 (2016).
11. Eden, E., Navon, R., Steinfeld, I., Lipson, D. & Yakhini, Z. GOrilla: a tool for discovery and visualization of enriched GO terms in ranked gene lists. *BMC bioinformatics* **10,** 48 (2009).
12. Schulz, M. H. *et al.* DREM 2.0: Improved reconstruction of dynamic regulatory networks from time-series expression data. *BMC systems biology* **6,** 104 (2012).
13. Szklarczyk, D. *et al.* STRING v10: protein–protein interaction networks, integrated over the tree of life. *Nucleic acids research* **43,** D447–D452 (2014).
14. Consortium, G. O. *et al.* Expansion of the Gene Ontology knowledgebase and resources. *Nucleic acids research* **45,** D331–D338 (2017).
15. Smith, C. L. *et al.* Mouse Genome Database (MGD)-2018: knowledgebase for the laboratory mouse. *Nucleic acids research* **46,** D836–D842 (2017).
16. Mouse Genome Informatics, T. J. L. *Mouse Genome Database (MGD)* `http://www.informatics.jax.org` (2017).
17. LeCun, Y., Bottou, L., Bengio, Y. & Haffner, P. Gradient-based learning applied to document recognition. *Proceedings of the IEEE* **86,** 2278–2324 (1998).
