# Supplementary material for: A web server for comparative analysis of single-cell RNA-seq data
Source: Nat Commun. 2018 Nov 13;9:4768. doi: 10.1038/s41467-018-07165-2 (PMC6233170; doi:10.1038/s41467-018-07165-2)
Supplement: Supplementary file 3 — Description of Additional Supplementary files [file 41467_2018_7165_MOESM3_ESM.pdf]

Source data for all applicable figures (Figure 3)
